# Endophytic fungi from two isolated *Bethencourtia palmensis* populations*:* metabolomic and bioactivity fingerprinting

**DOI:** 10.1002/ps.71108

**Published:** 2026-07-17

**Authors:** Jorge Rojas López‐Menchero, Juan Imperial, Carmen Elisa Díaz, María Fe Andrés, Azucena González‐Coloma

**Affiliations:** ^1^ Instituto de Ciencias Agrarias ICA‐CSIC Madrid Spain; ^2^ Centro de Biotecnología y Genómica de Plantas (CBGP, UPM‐INIA/CSIC), UPM Campus de Montegancedo Madrid Spain; ^3^ Instituto de Productos Naturales y Agrobiología IPNA‐CSIC Tenerife Spain

**Keywords:** *Bethencourtia palmensis*, Canary Islands, fungal endophytes, chemical fingerprint, biological activity, biopesticides

## Abstract

**BACKGROUND:**

*Bethencourtia palmensis* (Nees) Choisy is an endemic plant of the Canary Islands, with different populations on La Palma and Tenerife, that contains biopesticidal compounds and whose endophytic fungi have also shown potential for biopesticide production. This study explores the metabolic and biopesticide potential of endophytic culturable fungi isolated from two island populations of *Bethencourtia palmensis* [Tenerife (T) and La Palma (P)].

**RESULTS:**

The two plant populations showed distinct chemical profiles: 11β‐acetoxy‐5α‐(angeloyloxy)silphinen‐3‐one and tetratriacontane were major components in T, while 5α‐senecioyloxy‐silphinen‐3‐one, tetratriacontane, and jacaranone were predominant in P. From potato dextrose agar (PDA) medium, 21 and 16 fungal isolates were obtained from T and P, respectively. Ethyl acetate extracts were tested against *Myzus persicae*, *Meloidogyne javanica*, *Botrytis cinerea*, and *Fusarium verticillioides*. Three isolates from T (14%), belonging to *Aspergillus* and *Penicillium*, showed bioactivity. In contrast, 13 isolates from the P population (81%) were bioactive and belonged to *Alternaria*, *Aureobasidium*, *Aspergillus*, *Penicillium*, and *Stemphylium*. Additional isolation from P using YES medium (2% yeast extract, 15% sucrose, 1.5% agar) yielded 31 new isolates, 20 of which (65%) were bioactive, including genera not recovered on PDA. Gas chromatography–mass spectrometry‐based metabolite fingerprinting revealed distinctive patterns associated with specific genera.

**CONCLUSION:**

The findings highlight significant differences in the plant chemistry and bioactive endophytes between two island populations of *B. palmensis*. Furthermore, these strains are a promising source of bioactive compounds. The diverse range of bioactivities observed suggests high potential for the discovery of novel biopesticides with applications in sustainable agriculture. © 2026 The Author(s). *Pest Management Science* published by John Wiley & Sons Ltd on behalf of Society of Chemical Industry.

## Graphical abstract

We compared two island populations of *Bethencourtia palmensis* and their culturable endophytic fungi, revealing distinct plant chemistries and a higher biopesticidal potential in the endophytic fungi from La Palma population along with high potential for the discovery of novel compounds.

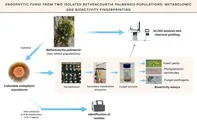

## 1 INTRODUCTION

Endophytic fungi are microorganisms that live within plant tissues without causing harm to their host.^1^ In many cases, they confer beneficial effects, such as enhancing plant fitness or promoting resistance to biotic and abiotic stresses. These fungi are emerging as a major source of new bioproducts with promising applications in medicine, agriculture, and the food industry.^2^ Despite this potential, most fungal endophytes remain understudied. Estimates of their global diversity range between 1 and 3 million species.^3^ However, to date, only 204 924 species have been formally catalogued (https://www.speciesfungorum.org; accessed 21 January 2026). This gap between what is estimated to exist and what has been really described highlights the urgency of continued exploration, particularly in environments associated with high biodiversity, ecological isolation, and chemical novelty, especially as the discovery of truly novel natural products is becoming increasingly rare.^4^

Among potential reservoirs for novel endophytic fungi, endemic plants are especially attractive targets. Their unique phytochemistry and restricted distribution make them likely to harbor distinct and underexplored microbial communities. Targeted exploration of these systems can lead to the discovery of new species or strains with specialized bioactivities as demonstrated in recent studies focused on agricultural^5^ or medical^6^ applications. In crop protection, these microorganisms have been applied either as biocontrol agents (whole organisms) or natural product‐based biopesticides. Endophytes can produce a wide array of secondary metabolites with application as biopesticides.^7^

*Bethencourtia palmensis* (Nees) Choisy is an endemic plant species present in the islands of Tenerife and La Palma (Canarian Archipelago, Spain). A study based on molecular analyses revealed significant genetic divergence between the Tenerife and La Palma populations of *B. palmensis*, which are morphologically similar. This was explained by the ocean acting as a natural barrier, leading to lower levels of gene flow between islands.^8^ Although morphological traits are similar between both plant populations, their chemistry and the composition of their endophytic communities may be different due to environmental effects.^9^, ^10^ The chemistry of *B. palmensis* (Tenerife population) has been extensively studied due to its unique content in silphinene sesquiterpenes with strong insect antifeedant effects,^11^, ^12^ but the chemistry of *B. palmensis* from La Palma has not been studied. The endophytic mycobiota of *B. palmensis* from Tenerife has been partially explored, and the metabolic potential of two of the isolates, both of them *Aspergillus* strains, demonstrated by the isolation of antifungal, acaricidal,^5^, ^13^ and nematicidal (Patent No. ES2920198 B2) compounds. However, a complete description of the mycobiota from *B. palmensis* has not been yet reported.

In this study, the qualitative and quantitative differences in silphinene sesquiterpene content between two populations of *B. palmensis* from Tenerife and La Palma were analyzed. A screening of culturable fungal endophytes from the two populations was conducted, and their ability to produce bioactive extracts (insecticidal, nematicidal, or antifungal activity) evaluated. The bioactive isolates were identified taxonomically via Internal Transcribed Spacer (ITS) BD Difco™, Sparks, USA sequencing, and comparative metabolite fingerprinting was performed to assess endophytic chemical diversity.

## 2 MATERIAL AND METHODS

All procedures that required it were carried out under sterile conditions.

### 2.1 Biological material

*Bethencourtia palmensis* aerial plant parts were collected at flowering season from their natural habitats in Tenerife and La Palma (Canary Islands, Spain). Voucher specimens have been deposited at the herbarium of the Botanical Garden, La Orotava, Tenerife, Spain (voucher number ORT 36393). In Tenerife, *B. palmensis* was collected in Barranco del Río (Arico, Tenerife) (28°11′26″ N, 16°29′52″ W), 1400 m altitude. In La Palma, two specimens of *B*. *palmensis* were collected in Caldera de Taburiente National Park (28°45′38.62″ N, 17°52′24.51″ W and 28°44′56″ N, 17°50′54.18″ W at 2285 and 2271 m altitude, respectively). La Palma specimens were collected and identified by Ángel Palomares (Park Director) and Tenerife specimens were identified by Dr. A. Santos. Plants were identified based on their morphological characters.

### 2.2 Endophyte isolation

Stem, flowers, and leaves were used for the isolation. Plant surfaces were washed and then surface sterilized by immersion in EtOH 75% for 1 min, followed by immersion in NaClO 1% for 2 min. Samples were then thoroughly rinsed in three successive washes of sterile distilled water to remove residual sterilizing agents. The effectiveness of surface sterilization was verified by plating the final rinse water on potato dextrose agar (PDA; BD Difco, USA) to confirm the absence of microbial growth.

Equal pieces of each organ were cultured on PDA or YES (2% yeast extract; 15% sucrose; 1.5% agar) plates, both media containing 50 mg L^−1^ of chloramphenicol to inhibit bacteria. In additiony, to obtain more isolates, stem samples were crushed in sterile water with a mortar and pestle, and both solid and liquid fractions were cultured.

As soon as fungi started to spread on the plates, they were serially subcultured onto new plates for purification. Isolates from T populations had been obtained previously and were given a ‘SP’ identifier. Isolates from the P populations were given a ‘BP’ identifier, followed by ‘H,’ ‘F,’ ‘T’, ‘M,’ or ‘G,’ for leaf, flower, stem, crushed stem, or crushing supernatant, respectively, to indicate their origin.

### 2.3 Molecular identification of isolates

Molecular identification of selected bioactive isolates was carried out through PCR amplification of the ITS region and Sanger sequencing.

To that end, DNA was extracted using a modified CTAB method^14^ as follows: mycelium was incubated for 3 days in potato dextrose broth (PDB; Difco) at 25 °C, washed with distilled water, and centrifuged at 16 000×*g* for 1 min. Portions of approximately 100 mg of mycelium were ground to powder in liquid nitrogen. Ground mycelia were suspended in 500 μL of CTAB buffer (2% CTAB, 1.4 M NaCl, 100 mM Tris–HCl, 20 mM EDTA, pH 8), 1.2 μL 2‐mercaptoethanol and 2 μL RNase A (10 mg mL^−1^) per extraction tube, and incubated at 65 °C for 30 min, with periodic inversion every 5 min. Proteins and lipids were removed with 500 μL of chloroform‐isoamyl alcohol (24:1), mixed gently, and centrifuged at 17 300×*g* for 10 min. The aqueous phase was transferred to a clean tube and this step was repeated once again. DNA was precipitated with 550 μL of cold isopropanol, mixed gently, and incubated at −20 °C for 15 min. Following centrifugation at 9500×*g* for 10 min at 4 °C, the pellet was washed with 500 μL of 100% ethanol, centrifuged again, and the process was repeated with 70% ethanol. Residual ethanol was removed and the pellet dried at 40 °C for 15 min. The DNA pellet was resuspended in 100 μL of milli‐Q water and stored at −20 °C.

PCR amplification was carried out using 1 μL of the extracted genomic DNA solution as template and DreamTaq polymerase (Thermo Fisher Scientific, Waltham, USA) in a thermocycler. with an initial denaturation step at 95 °C for 3 min, followed by 35 cycles of denaturation at 95 °C for 30 s, annealing at 55 °C for 30 s, and extension at 72 °C for 30 s. A final extension step was carried out at 72 °C for 5 min to ensure complete termination of amplified sequences. The primers used for this reaction were, for the SP isolates, ITS1 (5′‐TCCGTAGGTGAACCTGCGG‐3′) and ITS4 (5′‐TCCTCCGCTTATTGATATGC‐3′), and, for the BP isolates, ITS3 (5′‐GCATCGATGAAGAACGCAGC‐3′) and ITS4.^15^

Correct amplification was confirmed by agarose gel electrophoresis; bands were excised from gels using NucleoSpin® Gel and PCR Clean‐up kit (Takara Bio, San Jose USA) and outsourced for Sanger sequencing. Forward and reverse sequences were aligned with BioEdit (v. 7.2.5), trimming unaligned ends. Base calls were manually curated against chromatograms and consensus sequences derived for BLAST (https://blast.ncbi.nlm.nih.gov) comparison to publicly available databases for taxonomic identification of isolates. Finally, basic morphological properties of isolates were compared to those described for their assigned taxonomic entity.

ITS sequences were deposited in GenBank. Accession numbers are listed in Supporting Information, Table S2.

### 2.4 Extract preparation

#### 2.4.1 Plant extracts

*B. palmensis* aerial parts were dried in an air‐forced oven at 30 °C and ground. Dry materials were extracted with EtOH by maceration for 3 days at room temperature and the solvent was evaporated at reduced pressure to give crude extracts.

#### 2.4.2 Fungal extracts

For each fungal isolate, mycelia were scrapped from fresh cultures on PDA plates and transferred to three 100‐mL flasks containing 50 mL of PDB (Difco) for La Palma isolates or modified Czapek Dox (per liter: glucose 60 g, yeast extract 0.1 g, KH_2_PO₄ 0.5 g, NaNO₃ 0.2 g, MgSO₄·7H_2_O 0.05 g, FeSO₄·7H_2_O 1 mg, ZnSO₄·7H_2_O 3 mg) for Tenerife isolates for batch fermentation (120 rpm at 25 °C) for 21 days or 14 days, respectively. After this period, all flasks were checked for contamination and culture media were separated from mycelia by Büchner vacuum filtration.

Filtrates were liquid/liquid extracted with ethyl acetate (1:1) three times to maximize metabolite extraction. For each isolate, pooled organic phases were mixed with sodium sulphate to remove water traces, filtered, and dried in a rotary evaporator at 40 °C under a vacuum of 240 mbar. Dried extracts were weighed and kept dry at 4 °C.

### 2.5 Chromatographic analysis

Gas chromatography–mass spectrometry (GC–MS) was carried out on a Shimadzu GC‐2010 gas chromatograph coupled to a Shimadzu GCMS‐QP2010 Ultra mass detector (Shimadzu, Kyoto, Japan) with a single quadrupole analyzer, electron impact ionization source at 70 eV, helium as the carrier gas, and equipped with a 30 × 0.25 mm internal diameter and 0.25‐μm phase thickness capillary column (Teknokroma TRB‐5, 5% phenyl, 95% dimethyl polysiloxane for plant extracts, or MEGA‐5 MS 5% phenyl, 95% methyl polysiloxane for fungal extracts, Sant Cugat del Vallès, Spain).

Working conditions: Dried extracts were dissolved in dichloromethane (4 mg mL^−1^ DCM), filtered and analyzed by GC–MS. Portions of 1 μL were injected in split mode (split ratio 20/1) using a Shimadzu AOC‐20i automatic injector, injector temperature 300 °C, temperature of the transfer line connected to the mass spectrometer 250 °C, and temperature of the ionization source 220 °C. For plant extracts, the initial column temperature was 70 °C, ramping up to 290 °C at 6 °C min^−1^, and running at 290 °C for 20 min. For fungal extracts, the initial column temperature was 110 °C, ramping up to 290 °C at 7 °C min^−1^, and running at 290 °C for 20 min.

A full scan with *m/z* range 35–450 was performed. Relative amounts of individual components were calculated based on the peak area without a correction factor. Retention data (the mass spectra and Kovats retention indexes) were used to assess compound identity by comparison with silphinene standards (silphinenes previously isolated in our laboratory from *B. palmensis*) and the Wiley 229 Mass Spectral Database.

Chemical profiles of each isolate were established based on their GC–MS analysis, considering the relative percentage of peak area for each compound. Compounds with a peak area above 1% were included, as well as those below 1% if they appeared above that threshold in any other isolate. Based on these data, isolates were grouped by hierarchical clustering according to their chemical profiles. Clustering was performed in R (v. 4.3.1) using Euclidean distance and the Ward.D2 method. The graphical representations of clustering results, including dendrograms and heatmaps, were generated using the R packages dendextend, heatmaply, and plotly.

### 2.6 Bioassays

Pictures of the bioassays are shown in Supporting Information, Fig. S1.

#### 2.6.1 Antifeedant activity

The *Myzus persicae* colony was reared on bell pepper (*Capsicum annuum;* Ramiro Arnedo, Calahorra, Spain) and maintained at 22 ± 1 °C, >70% relative humidity with a photoperiod of 16 h:8 h (light:dark) in a growth chamber.

Bioassays were conducted with 1.0‐cm^2^ leaf fragments of *C. annuum*. Test solutions (10 μL) were applied at initial doses of 10 mg mL^−1^ to the upper surface of leaf fragments.^16^ *M. persicae* apterous adults (24–48 h old) were placed in 20 (2 × 2 cm) ventilated plastic boxes (10 per box) and kept in a growth chamber for 24 h. Settling was measured to quantify antifeedant activity by counting the number of aphids on each leaf fragment. Settling inhibition (%SI) was calculated as [1 − (T/C) × 100], where T and C represent treated and control leaf fragments, respectively. The effects (%SI) were analyzed by the non‐parametric Wilcoxon signed‐rank test. Extracts were considered bioactive when they reached values >70% settling inhibition. The positive control for *M. persicae* was thymol (Sigma‐Aldrich, USA), with an EC_50_ of 7.6 g cm^−2^.

#### 2.6.2 Nematicidal assay

A population of the root‐knot nematode *Meloidogyne javanica* was maintained on tomato plants (*Lycopersicon esculentum* var. Marmande, Ramiro Arnedo, Calahorra, Spain) cultivated in pots under controlled conditions (25 ± 1 °C and relative humidity above 70%). Second‐stage juveniles (J2) that emerged within 24 h from egg masses manually extracted from infected tomato roots were selected for use in the assays. Bioassays were conducted in 96‐well microplates (Becton‐Dickinson, NJ, USA), following the procedure described by Andrés *et al*.^17^ Organic extracts were tested at 1 mg mL^−1^ concentration in each well. Juvenile mortality was assessed after 72 h. Each treatment was performed in quadruplicate. Mortality percentages were calculated and corrected using the Schneider–Orelli formula. Extracts were considered bioactive when mortality rates were >90%.

#### 2.6.3 Antifungal assays

*Botrytis cinerea* was obtained from the fungal collection at Instituto de Productos Naturales y Agrobiologia‐CSIC. The spore stock was frozen and maintained at −80 °C in 20% glycerol.

For BP isolates, spore germination inhibition assays were conducted using 96‐well plates as described.^18^ Each well (four replicates) contained 100 μL of Roswell Park Memorial Institute 1640 medium (RPMI 1640), 20 μL of spores (10^7^ spores mL^−1^), and 80 μL of water with 2.5% of a Dimethyl Sulfoxide (DMSO) solution (80 mg mL^−1^ of extract in DMSO). Plates were then incubated at 25 °C for 24 h.

After incubation, 25 μL of a solution containing 3‐(4,5‐Dimethylthiazol‐2‐yl)‐2,5‐Diphenyltetrazolium Bromide (MTT,5 mg mL^−1^) and menadione (1 mM) (Sigma‐Aldrich, St. Louis, USA) was added to each well and incubated for 3 h. After removal of the supernatant, 200 μL of acid isopropanol (containing 5% HCl) was added to each well and incubated for 30 min. Changes in medium absorbance (490 nm) were recorded with an ELx800 absorbance microplate reader (Biotek, USA) using Biotek Gen5 2.01 software to assess fungal growth inhibition.

For SP isolates, extracts were tested against *Fusarium verticillioides* following the protocol described in Morales‐Sánchez *et al*.^5^ Assays were carried out in 12‐well cell plates. Extract solutions dissolved in EtOH were mixed with PDA medium (reaching a final concentration of 1 mg mL^−1^) and 0.05 mg mL^−1^ of MTT and added to each well of the plate. Subsequently, a 1‐cm diameter disk of *F. verticillioides* was placed at the center of the well. All treatments had four replicates and EtOH was used as a negative control. After an incubation period of 48 h in the dark at 28 °C, or until the mycelium reached a diameter of 2 cm, mycelial growth was measured using ImageJ software (http://imagej.nih.gov/ij/). Bioactivity was expressed as a percentage of mycelium growth inhibition. Extracts were considered bioactive when fungal growth was inhibited >70%.

## 3 RESULTS

### 3.1 Chemical analysis of the plants

Chemical analysis (GC–MS) of plant extracts from both T and P *B. palmensis* populations showed important differences in their chemical composition (Fig. 1). The T population showed 11β‐acetoxy‐5α‐(angeloyloxy)silphinen‐3‐one (**3**, 29.0%) and tetratriacontane (**4**, 16.0%) as the major components, while the P population contained jacaranone (**1**, 19.8%), 5α‐senecioyloxy‐silphinen‐3‐one (**2**, 37.7%), and tetratriacontane (**4**, 29.7%) as major components. The full composition table is included in Supporting Information, Table S3.

**Figure 1:**
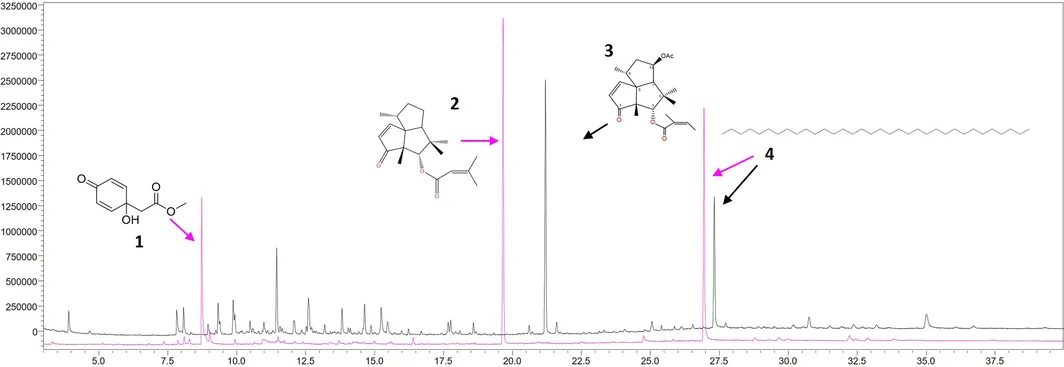
GC–MS chromatograms showing the chemical profiles of La Palma (pink) and Tenerife (black) extracts. Compounds: jacaranone (**1**), 5α‐senecioyloxy‐silphinen‐3‐one (**2**), 11β‐acetoxy‐5α‐(angeloyloxy)silphinen‐3‐one (**3**), and tetratriacontane (**4**).

### 3.2 Endophytic fungal isolates

Endophytic fungi from *B. palmensis* were isolated in PDA plates. From the Tenerife population, a total of 21 SP isolates were obtained and only three of them (14%) showed relevant bioactivity (>70% against insects and fungi, >90% against nematodes). These were characterized as *Aspergillus* sp. (SPH2 and SPH9, both isolated from stems) and *Penicillium* sp. (SPH17, isolated from leaves). From the La Palma population, a total of 16 BP isolates were obtained and 13 were active against at least one target (81% bioactive strains). They belonged to five genera: *Alternaria*, *Aureobasidium*, *Aspergillus*, *Penicillium* and *Stemphylium*). *Aspergillus* and *Penicillium* species appeared in both populations, while *Alternaria*, *Aureobasidium*, and *Stemphylium* were only found in the La Palma population among the bioactive isolates (Fig. 2).

**Figure 2:**
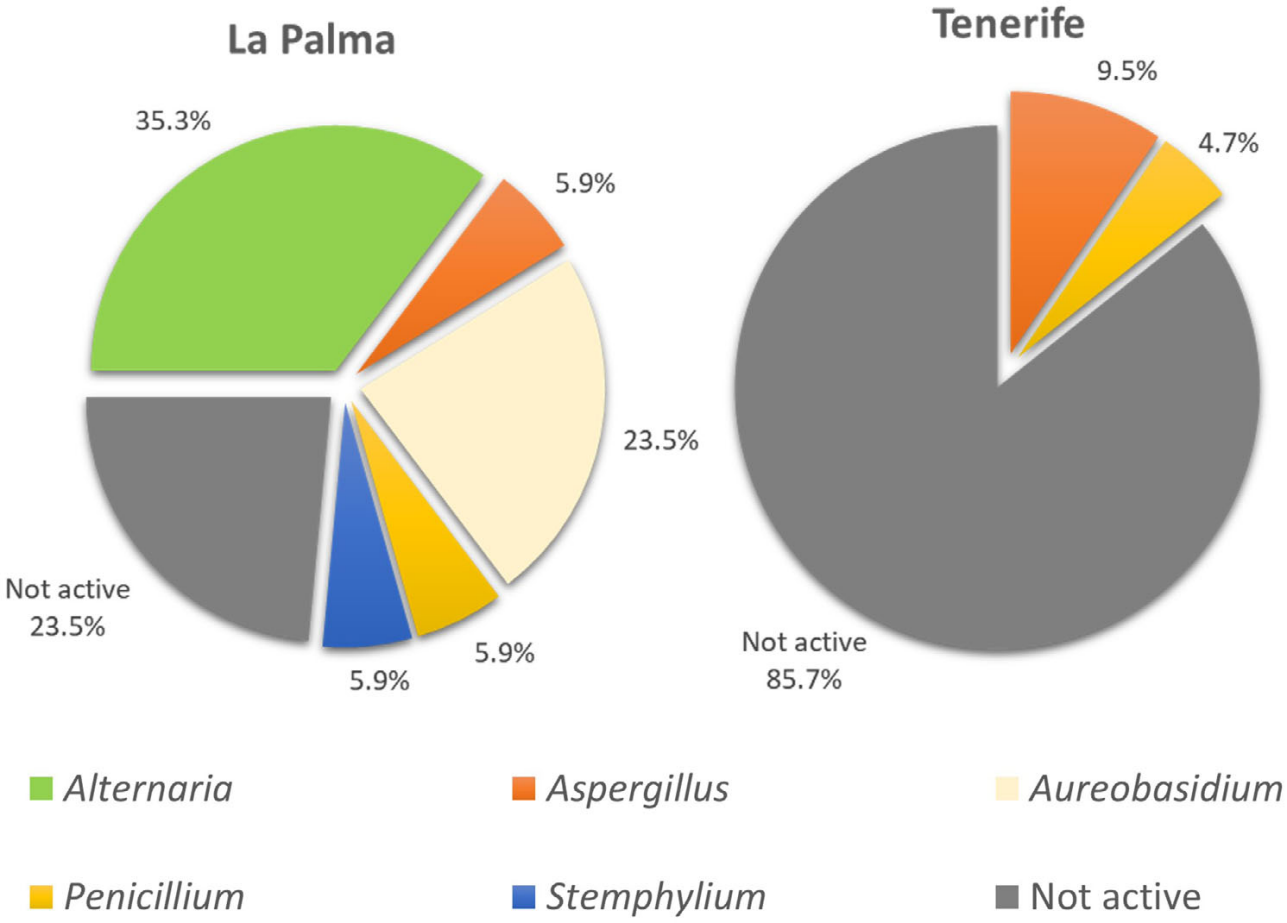
Comparison of potato dextrose agar fungal isolates from La Palma and Tenerife *B. palmensis* populations.

The striking abundance and diversity of bioactive isolates found in the BP La Palma population prompted us to undertake an additional isolating effort for this population. Using YES medium for isolations, 31 new isolates were obtained, out of which 20 (65%) were bioactive (Table 1). Overall, slightly more isolates with bioactivity were obtained on YES than on PDA medium (16 PDA, 20 YES), but with a clear media selectivity for certain taxa, such as *Alternaria* (6 PDA/2 YES) and *Aureobasidium* (3 PDA/1 YES), preferentially isolated on PDA, *Aspergillus* (3 PDA/9 YES), or *Stemphyllium* (1 PDA/2 YES), preferentially isolated on YES. *Penicillium* did not show any difference (2 PDA/2 YES), while strains from *Cladosporium*, *Epicoccum*, *Phoma*, and *Mucor* (the only isolate outside phylum Ascomycota) were only isolated on YES. Fermentation yields (Table 1) were strain dependent, but some genera, such as *Aspergillus*, showed consistently higher yields with two strains, BPT13 and SPH9 showing the highest yields.

**Table 1:** Bioactive fungal isolates from *B. palmensis*

Population	Isolate	Culture medium	Yield (mg in 100 mL)	ITS identification	Antifeedant activity (%)	Nematicidal activity (% mortality)	Antifungal activity* (%)
					100 μg cm^−2^	1 mg mL^−1^	800 μg mL^−1^
P	BPF4.1	PDA	5.9	*Alternaria* sp.	88.2 ± 8.3	99.7 ± 0.3	79.5 ± 1.3
P	BPF5	PDA	4.5	*Alternaria* sp.	64.9 ± 9.7	100 ± 0	77.1 ± 2.4
P	BPF6	PDA	3.5	*Alternaria* sp.	70.9 ± 8.2	0 ± 0	86.3 ± 0.4
P	BPT2	PDA	2.9	*Alternaria* sp.	92.9 ± 1.9	94.9 ± 0.6	46.4 ± 7.5
P	BPT3	PDA	5.6	*Alternaria* sp.	60.8 ± 9.6	15.0 ± 3.0	70.1 ± 2.3
P	BPT4	PDA	5.0	*Alternaria* sp.	49.2 ± 9.0	100 ± 0	79.8 ± 1.7
P	BPF7	YES	4.6	*Alternaria* sp.	43.3 ± 7.8	16.8 ± 1.1	73.6 ± 2.0
P	BPH1	YES	4.3	*Alternaria* sp.	57.5 ± 9.5	8.6 ± 1.9	77.6 ± 1.2
T	SPH2	PDA	33.1	*Aspergillus* sp.	52.2 ± 8.0	25.0 ± 5.0	81.8 ± 1.6
T	SPH9	PDA	55.4	*Aspergillus* sp.	59.5 ± 7.1	100 ± 0	53.9 ± 4.5
P	BPT13	PDA	47.0	*Aspergillus* sp.	73.1 ± 8.6	100 ± 0	29.9 ± 3.0
P	BPG5	YES	7.2	*Aspergillus* sp.	80.3 ± 4.9	96.8 ± 1.1	48.9 ± 3.2
P	BPG6.2	YES	24.5	*Aspergillus* sp.	90.8 ± 3.5	100 ± 0	25.7 ± 4.2
P	BPH6	YES	25.9	*Aspergillus* sp.	78.2 ± 7.8	100 ± 0	33.3 ± 4.3
P	BPM1	YES	24.4	*Aspergillus* sp.	94.9 ± 2.2	100 ± 0	19.2 ± 15.2
P	BPM2	YES	19.9	*Aspergillus* sp.	79.0 ± 4.5	100 ± 0	23.2 ± 4.2
P	BPM3	YES	24.7	*Aspergillus* sp.	88.1 ± 7.0	91.6 ± 4.7	53.6 ± 3.6
P	BPM5	YES	22.7	*Aspergillus* sp.	96.6 ± 1.6	100 ± 0	0 ± 0
P	BPM7.1	YES	17.8	*Aspergillus* sp.	48.3 ± 12.1	100 ± 0	37.8 ± 4.5
P	BPM9.2	YES	24.1	*Aspergillus* sp.	86.0 ± 4.4	100 ± 0	23.9 ± 4.4
P	BPH3	PDA	15.2	*Aureobasidium* sp.	78.2 ± 6.3	10.1 ± 2.0	64.6 ± 3.7
P	BPH5	PDA	10.7	*Aureobasidium* sp.	73.1 ± 10.9	14.4 ± 1.9	75.3 ± 1.5
P	BPT1	PDA	10.8	*Aureobasidium* sp.	72.5 ± 7.9	6.6 ± 3.3	45.5 ± 1.6
P	BPT15	YES	6.3	*Aureobasidium* sp.	66.5 ± 9.6	0.5 ± 0.8	74.5 ± 1.9
P	BPM13	YES	22.1	*Cladosporium* sp.	72.1 ± 9.0	1.9 ± 1.2	78.4 ± 1.0
P	BPF3	YES	6.0	*Epicoccum* sp.	95.8 ± 1.1	13.8 ± 4.5	0 ± 0
P	BPG3	YES	7.7	*Mucor* sp.	90.7 ± 5.6	8.0 ± 1.8	57.2 ± 4.7
T	SPH17	PDA	32.2	*Penicillium* sp.	78.8 ± 8.8	52.4 ± 7.3	16.6 ± 9.5
P	BPH4	PDA	2.8	*Penicillium* sp.	79.6 ± 7.9	4.2 ± 0.6	79.1 ± 1.1
P	BPG2	YES	3.5	*Penicillium* sp.	88.9 ± 6.1	10.6 ± 0.9	61.6 ± 1.7
P	BPG6.1	YES	5.1	*Penicillium* sp.	46.6 ± 8.6	18.5 ± 2.7	80.2 ± 0.5
P	BPT11	YES	2.8	*Phoma* sp.	29.8 ± 8.5	15.0 ± 2.1	77.9 ± 0.4
P	BPF8	PDA	9.7	*Stemphylium* sp.	14.8 ± 6.2	100 ± 0	38.1 ± 3.2
P	BPF9	YES	5.0	*Stemphylium* sp.	78.7 ± 9.3	17.6 ± 2.7	23.4 ± 8.5
P	BPT9	YES	2.8	*Stemphylium* sp.	74.6 ± 6.8	97.6 ± 0.6	73.3 ± 1.6

Extract yield (mg extract per 100 mL medium), ITS‐based identification at genus level and bioactivity (mean ± standard error) against *M. persicae* (% settling inhibition), *M. javanica* (% mortality), and pathogenic fungi (*Botrytis cinerea*/*Fusarium verticillioides*. La Palma (P) and Tenerife (T) isolates were fermented in PDB and modified Czapek‐Dox, respectively. Extracts were considered bioactive above a 70% threshold.

* Fungal targets: *Botrytis cinerea* spore germination for La Palma isolates, *Fusarium verticillioides* mycelial growth for Tenerife isolates.

### 3.3 Chemical profiling of endophytic isolates

The extracts from isolates displaying bioactivity (Table 1) were analyzed by GC–MS and clustered based on their chemical profiles (Fig. 3 and Supporting Information, Table S1). Additionally, activity data were categorized as active (bioactivity >70%), moderately active (40–70%), or not active (<40%). Bioactive extracts were grouped into five major clusters (A–E) and two subclusters (Ef, Eg) with two outgroups (Ef1 and Ef2, Eg1 and Eg2). A total of 191 different compounds were detected, with 87 remaining unidentified (<80% similarity with the database), indicating a high number (45%) of potentially undiscovered compounds. The presence of unidentified compounds varied among clusters: A (0%), B (36.6%), C (21.4%), D (40.7%), and E (33.6%).

**Figure 3:**
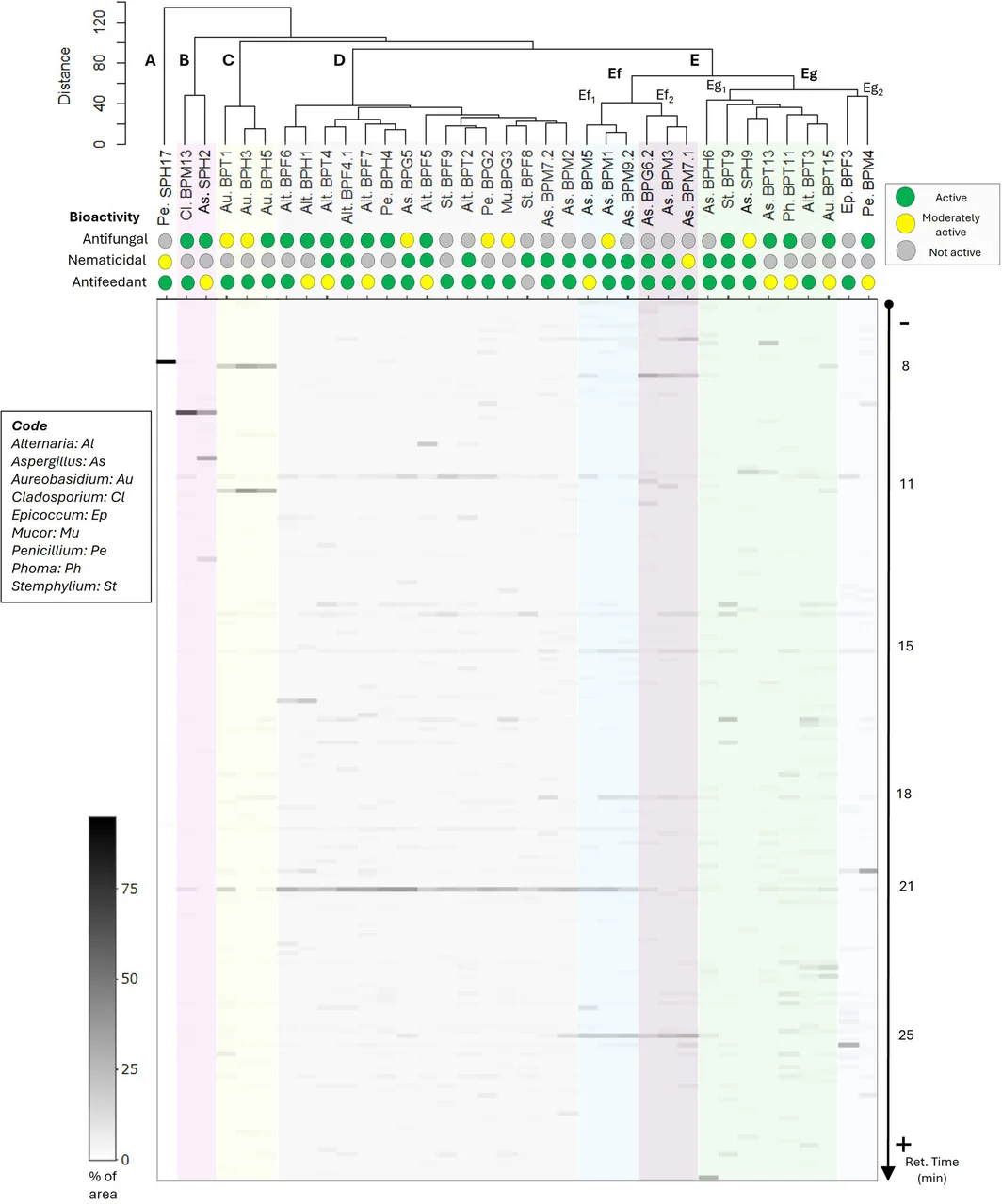
Hierarchical clustering of extract chemical profiles from *B. palmensis* fungal isolates determined by GC–MS. Bioactivities against different targets of each extract are represented by circles colored in green (active) yellow (moderately active), and grey (not active). Relative abundances of compounds are represented by a greyscale heatmap and arranged unidimensionally as bands along a retention time scale. Heatmap displays compounds **1**–**191** (numbers not shown in the *y* axis) between retention times 3.406 and 37.597 min.

#### 3.3.1 Cluster A

Formed exclusively by *Penicillium* strain SPH17, characterized by the exclusive production of compound **15** Retention Time (RT) 7.676, penicillic acid, that showed antifeedant activity.

#### 3.3.2 Cluster B

*Cladosporium* BPM13 and *Aspergillus* SPH2 strains characteristically produced compound **25** (RT 9.093, mullein, 66.6% and 32% abundance, respectively), with antifungal (both strains) and antifeedant (BPM13) activities.

#### 3.3.3 Cluster C

Composed exclusively of *Aureobasidium* strains (BPT1, BPH3, and BPH5), it is characterized by compounds **15** (RT 7.940, massoia lactone 27.5% average abundance) and **42** (RT 11.622, 3,5‐dihydroxydecanoic acid δ‐lactone, 31.5% average abundance), with activity against *M. persicae* (all) and *B. cinerea* (BPH5).

#### 3.3.4 Cluster D

In this cluster, *Alternaria* is the predominant genus (BPF6, BPH1, BPT4, BPF4.1, BPF7, BPF5, BPT2), followed by *Aspergillus* (BPG5, BPM7.2, BPM2), *Stemphylium* (BPF9, BPF8), and *Penicillium* (BPH4, BPG2). It is characterized by compounds **39** (RT 11.434, acrylic acid tetradecanyl ester, 6.64% average abundance) and **129** (RT 20.856, oleamide, 27.63% average abundance), and includes antifungal (all the *Alternaria* strains, except BPT2, and the *Penicillium* BPH4), nematicidal (*Alternaria* strains BPT4, BPF4.1, BPF5, and BPT2; *Aspergillus* strains BPG5, BPM7.2 and BPM2; *Stemphylium* sp. BPF8), and antifeedant (*Alternaria* strains BPF6, BPF4.1, and BPT2; *Aspergillus* strains BPG5, BPM7.2, and BPM2; *Penicillium* strains BPH4 and BPG2; Mucor sp. BPG3; *Stemphylium* BPF9).

#### 3.3.5 Cluster E

Ef: Composed exclusively of *Aspergillus* isolates (Ef1: BPM5, BPM1, BPM9.2) and (Ef2: BPG6.2, BPM3, BPM7.1) is separated into Ef1 and Ef2 by differences in compound **17** (RT 8.026 min, 2‐(4‐hexyl‐2,5‐dioxofuran‐3‐yl)acetic acid, 3.11% average in Ef1 *vs* 17.56% average in Ef2), and characterized by **160** (RT 25.807 min, 3,6‐dibenzylpiperazine‐2,5‐dione, 17.38% average). The strains were mostly active on *M persicae* (all of them, except BPM5) and *M. javanica* (all of them, except BPM7.1).

Eg: Composed of Eg1 (BPH6, BPT9, SPH9, BPT13, BPT11, BPT3, BPT15) and Eg2 (BPF3, BPM4), a heterogeneous group in both chemical profile and bioactivity.

Eg1: BPH6 is an *Aspergillus* isolate and characterized by **191** (RT 37.597 min, stearyl palmitate 28.79%). *Stemphylium* sp. BPT9 is characterized by **92 (**RT 16.989 min, oleyl alcohol, 21.0%) and **67** (14.401 min, C15‐C19 alkane, 15.0%). *Aspergillus* strains SPH9 and BPT13 have **38** (11.341 min, 9‐hydroxyhexyl‐itaconic acid methyl ester, 14.9% and 8.0%, respectively). In addition, BPT13 is characterized by **10** (RT 6.167 min, succinic acid, butyl ethyl ester, 20.1%) and BPT3 with **67** (RT 14.401 min, C15–C19 alkane, 6.18%), **92** (RT 16.989 min, oleyl alcohol, 13.5%), **93** (RT 17.073, 2,4‐ditert‐butyl‐6‐(1‐phenylethyl)phenol, 8.0%). *Aureobasidium* sp. strain BPT15 is characterized by **15** (RT 7.940, massoia lactone, 7.4%) and **42** (RT 11.622, 3,5‐dihydroxydecanoic acid δ‐lactone, 5.4%). The most active strains were *Stemphylium* sp. BPT9 (on all targets), *Aspergillus* strains BPH6 and SPH9 (nematicidal and antifeedant), followed by *Aspergillus* sp. BPT13, *Phoma* sp. BPT11, *Aureobasidium* sp. BPT15 (antifungal), and *Aspergillus* sp. BPT13 (antifeedant).

Eg2: Composed of *Epicoccum* strain BPF3 and *Penicillium* strain BPM4, with **125** as common differential compound (RT 19.608, tributyl acetylcitrate, 22.15% average), with individual major compounds **162** (RT 26.171, squalene, 32.05%) in *Epicoccum* BPF3 and **23** (RT 9.009, 4‐hydroxyphenylacetic acid, 8.19%) in *Penicillium* BPM4. These strains were antifeedant (BPF3) or antifungal (BPM4).

Based on the ITS identification and the chemical profiles obtained for the two dominant genera, *Aspergillus* and *Alternaria*, as well as other less prevalent ones, such as *Aureobasidium* and *Stemphylium*, the results suggest that not all isolates represent distinct strains. It is likely that some of them correspond to the same organism isolated multiple times during the process. For example, most *Aspergillus* isolates—except SPH2, SPH9, and BPM7.1—share the same bioactivity pattern (antifeedant and nematicidal), which could indicate repeated isolation of a single strain. In contrast, *Alternaria* isolates do not display such a consistent pattern, which may reflect the presence of different species or subspecies within the genus.

## 4 DISCUSSION

Clear metabolic differences between two *B. palmensis* populations from La Palma and Tenerife islands were observed. The Tenerife *B. palmensis* extract had a metabolic profile dominated by 11β‐acetoxy‐5α‐(angeloyloxy)silphinen‐3‐one, a compound with reported bioactivity against *Leptinotarsa decemlineata* and previously isolated from this population as the major component.^19^ The La Palma extract showed a different profile, characterized by the presence of the 5α‐senecioyloxy‐silphinen‐3‐one,^12^, ^20^ along with jacaranone, previously described in another *Bethencourtia* species (*B. hermosae*), and shown to have antifeedant and cytotoxic effects on insect and mammalian cell lines,^21^ and the alkane tetratriacontane, a kairomone‐like compound that favored parasitic/predatory activity of the biological control agents *Trichogramma chilonis* and *Chrysoperla zastrowi sillemi*.^22^ Previous studies reported that continental plant species often show higher investment in defensive traits than island plans (structural defenses and secondary metabolites), reflecting differences in herbivore pressure and evolutionary pressures.^23^ However, in this study the comparison involved two island populations (Tenerife and La Palma) with reported significant genetic divergence explained by a low gene flow across islands due to the ocean barrier.^8^

Higher biodiversity and bioactivity was found among endophytic fungal isolates from the La Palma *B. palmensis* population. Tenerife is a more ecologically diverse island, with steeper altitudinal gradients and older, more weathered soils. La Palma Island is geologically younger, with more recent lava flows, potentially hosting different microbial pools.^24^ In general, altitudinal and climatic variation leads to functional shifts in plant microbiota, with implications for nutrient cycling, stress tolerance, and defensive traits.^25^ The fact that plant populations across different islands harbor distinct fungal assemblages, even when grown under similar conditions, suggests that there is a host‐genotype‐driven component to microbial divergence.^26^ Other plant‐related factors, such as the host plant metabolome and host genotype/environment interactions, can also affect fungal colonization^27^, ^28^ and microbiota composition.^27^, ^29^, ^30^ In this case, besides environmental differences, metabolic changes observed between both populations could play a role in the plant endophytic community.

This work underscores the potential importance of endophyte screenings for plant protection traits. Among others, strains BPF3, BPF8, and BPG3, from La Palma, and SPH2, SPH9 from Tenerife, showed high potential for biocontrol applications. Our group has previously reported on metabolites from fungi isolated from endemic plants of the Canary Islands with potential applications in agriculture,^31^ such as *Phyllostictia capitalensis* (formerly *Guignardia magniferae*) isolated from *Persea indica*, with remarkable nematicidal activity,^32^ and two *Aspergillus* strains producing antifungal, acaricidal^5^, ^13^ and nematicidal (Patent No. ES2920198 B2) compounds.

The identification of bioactive fungal isolates has been carried out at the genus level on the basis of ITS sequence comparisons. Fungal taxonomic identification at species‐level resolution remains challenging due to the limited availability of high‐quality fungal genome assemblies and the inconsistent curation of public databases. In addition, the internal transcribed spacer, although widely used as a fungal barcode, often lacks the resolution needed to distinguish among closely related species. This limitation, coupled with potential annotation errors in repositories such as GenBank, has been shown to complicate accurate species assignment, particularly in metabarcoding.^33^ As a result, identifications based solely on ITS frequently remain at the genus level to avoid misidentifications. Our findings show that some isolates, especially the most abundant ones (i.e. *Aspergillus* with most isolates displaying a similar chemical and bioactivity profile), are likely the same species based on genetic, morphologic, and metabolic criteria. However, this hypothesis, based on distinct chemotypes and bioactivity profiles, should be confirmed through more comprehensive genetic analyses beyond ITS sequencing and morphological assessment. Deeper studies using multiple molecular markers, including protein‐coding genes,^34^ or even whole‐genome sequencing,^35^ would be necessary to determine the true genetic divergence among the isolated strains.

Some of our isolates were assigned to genera that are traditionally considered as pathogenic. However, our findings are consistent with endophyte screenings and strains reported in other studies. Members of *Alternaria* ^36^, ^37^ and *Aspergillus* ^38^, ^39^ are commonly identified as prevalent culturable species and have been previously reported as major producers of secondary metabolites.^40^, ^41^ *Aureobasidium*, another highly prevalent taxon, also appears frequently as an endophyte of various plants,^42^, ^43^ and some of these strains have yielded interesting bioproducts.^44^ Among the less abundant endophytes found here, there are notable examples in the literature of species with bioactive compounds that have potential applications in agriculture: *Epicoccum*,^45^ *Penicillium*,^46^ *Stemphylium*,^47^ and *Phoma*.^48^

Metabolic fingerprinting represents a complementary approach to help distinguish chemotypes within similar species and to identify fungal strains producing novel bioactive metabolites.^49^ Beyond taxonomic resolution, metabolome‐based analyses also offer a valuable strategy to pre‐select strains with distinctive chemical profiles for downstream analysis.^50^ In our study, metabolic fingerprinting revealed clear chemical divergence among several fungal isolates that appeared taxonomically related. These differences suggest the presence of distinct chemotypes within the same genus, with some isolates producing compounds not detected in closely related strains, even finding some strains able to produce potentially new compounds among them. This has been documented, especially in bacteria. Nephali *et al*. (2022) were able to reveal metabolic variation among *Bacillus* species, highlighting distinct patterns of compound production across different strains.^51^ In another example, Fiorini *et al*. (2022) analyzed 60 bacterial strains from diverse families and highlighted one particularly promising isolate that exhibited 107 unique MS features, including a cluster of 28 potentially novel compounds inferred from MS/MS similarity.^50^

Among the identified compounds in fermentation extracts, some have been described as bioactive or having industrial interest.

Cluster A

*Penicillium* SPH17 strain was characterized by the exclusive production of penicillic acid (**14**) with antifungal effects. This metabolite has been reported to have antimicrobial^52^ and antifungal^53^ activities, and also against the plant pathogenic oomycete *Pythium aphanidermatum*.^54^

Cluster B

*Cladosporium* BPM13 and *Aspergillus* SPH2, with antifungal and antifeedant activities, were characterized by mellein (**25**), reported as having acaricidal and antifungal activities.^5^

Cluster C

Cluster C is composed of *Aureobasidium* strains BPT1, BPH3, and BPH5, with activity against *M. persicae* and *B. cinerea*, and was characterized by massoia lactone (**15**) and 3,5‐dihydroxydecanoic acid δ‐lactone (**42**). Both compounds have been previously reported in this genus and might account for the observed antifungal activity, as the first one has been described as antifungal^55^ and the second one is structurally, and likely biosynthetically, related to the first one.^56^

Cluster D

Cluster D, dominated by *Alternaria* strains, several of them with antifungal activity, was characterized by acrylic acid tetradecanyl ester (**39**) and oleamide (**129**), none of them reported as bioactive to date. Strains BPF6 and BPH1 are separated from the other *Alternaria* isolates and were characterized by the presence of unidentified compound **88** (RT 16.328, base peak at *m*/*z* 181), a compound chemically exclusive to these two isolates within the genus, and the second most abundant metabolite in both strains, which makes it a potentially promising new compound.

Cluster E

Ef: Composed exclusively of *Aspergillus* isolates, it is characterized by 3,6‐dibenzylpiperazine‐2,5‐dione (**160**) and separated by differences in 2‐(4‐hexyl‐2,5‐dioxofuran‐3‐yl)acetic acid (**17**). Compound **160** has been reported to have antiproliferative activity against cancer cells.^57^ However, compounds derived from piperazines have been used as pesticides against fungi, insects or bacteria due to their low toxicity in mammals.^58^ On the other hand, compound **17** has been reported to have growth promotion effects in plants,^59^ antifungal effects against *Neurospora crassa*,^60^ and weak antibacterial activity.^61^ Interestingly, these extracts were not antifungal against the targets used in this work.

Eg: Eg1: Several of the compounds found in isolates from this cluster have potential interest as biopesticides. 9‐Hydroxyhexyl‐itaconic acid methyl ester (**38**), present in *Stremphyllium* BPT9 and *Aspergillus* SPH9, is a nematicidal agent (Patent No. ES2920198 B2), and succinic acid, butyl ethyl ester (**10**) has been reported as antibacterial.^62^ 2,4‐Ditert‐butyl‐6‐(1‐phenylethyl)phenol (**93**, reported with the code A 13–70 736) had antifertility effects on the fly of *Chochliomyia hominivorax*.^63^ BPT3 was chemically separated from the rest of the *Alternaria* strains. The presence of compounds **96** (not identified, RT 17.232, base peak at *m*/*z* 57), C34‐C36 alkane (**121**), and β‐sitosterol (**184**) suggests that BPT3 may represent a different chemotype or species.

The *Aureobasidium* strain BPT15 differs from other strains in cluster C, showing a lower content of massoia lactone (**15**) and 3,5‐dihydroxydecanoic acid δ‐lactone (**42**), compounds present in all *Aureobasidium* strains, while producing higher amounts of a C22–C36 alkane (**145**, 3.33% *vs* 0.26% average area), and also produces 2,4‐bis(1‐phenylethyl)phenol (**146**) and 2,4‐bis(dimethylbenzyl)‐6‐t‐butylphenol (**148**). This supports the idea that BPT15 may represent a different strain/chemotype within *Aureobasidium*. Compound **148** has been reported to exhibit both insecticidal and antifungal activity.^64^

Eg2: Composed of *Epicoccum* strain BPF3 and *Penicillium* strain BPM4, with tributyl acetlycitrate (**125**) as common differential compound, described as antifungal against *Curvularia verruculosa*.^65^ The individual major compounds were squalene (**162**) in BPF3 and 4‐hydroxyphenylacetic acid (**23**) in BPM4. Squalene is the central precursor for all phytosterols and a basic intermediate in the biosynthesis of sterols, hopanoids, and triterpenes as well as a key precursor in cholesterol production^66^ and known to have antimicrobial activity against Gram‐positive and Gram‐negative bacteria, and *Candida* species.^67^ Squalene also has potential as a bio‐based acaricide.^68^ 4‐Hydroxyphenylacetic acid has been reported as nematicidal^69^ and aphid antifeedant, similarly to the structurally related 2‐hydroxyphenylacetic acid.^70^

## 5 CONCLUSIONS

This study revealed significant chemical and microbiological differences between the two *B. palmensis* island populations studied. The La Palma population accumulated 5α‐senecioyloxy‐silphinen‐3‐one, while the Tenerife population produced 11β‐acetoxy‐5α‐(angeloyloxy)silphinen‐3‐one. These findings support the existence of a metabolic divergence between populations, in line with previous evidence of genetic differentiation between island populations.

Culturable endophytic fungi also differed notably between populations. PDA cultures from La Palma yielded a broader diversity of mostly bioactive isolates across more genera than those from Tenerife. Moreover, additional isolations using YES medium from the La Palma population increased both the number of bioactive strains and the recovery of new genera. This highlights that employing diverse isolation conditions could be relevant to maximize the retrieval of fungi with biotechnological potential.

Chemical profiling of fermentation extracts from the isolates showed distinct metabolite patterns associated with specific phylogenetic groups, supporting the presence of different strains or chemotypes within the dominant genera. These results reinforce the value of endophyte screening for the discovery of novel bioactive compounds and suggest a promising potential for their development as biopesticides in sustainable agriculture. Future optimization of fermentation conditions for the most promising strains will be essential to improve yields for scale‐up production and to identify specific molecules responsible for the observed bioactivities.

## CONFLICT OF INTEREST

The authors declare no competing interests.

## Supporting information


**Figure S1.** Pictures of bioactivity assays.

**Table S1.** Complete data set of GC–MS analysis of the fungal extracts from *Betehncourtia palmensis*.

**Table S2.** GenBank accession numbers and closest BLAST matches in NCBI for ITS sequences of bioactive *Bethencourtia palmensis* fungal isolates.

**Table S3.** Complete data set of GC–MS analysis of the plant extracts from two *Betehncourtia palmensis* populations (Tenerife and La Palma).

## Data Availability

Datasets generated in the current study are available from the corresponding author upon reasonable request. GenBank accession numbers for all strains isolated in this study are included in Supporting Information, Table S2.
