# Supplementary material for: Endophytic fungi from two isolated Bethencourtia palmensis populations: metabolomic and bioactivity fingerprinting
Source: Pest Manag Sci. 2026 Jul 17;82(12):11151–61. doi: 10.1002/ps.71108 (PMC13756819; doi:10.1002/ps.71108)
Supplement: Supplementary file 1 [file PS-82-11151-s001.docx]

**Supplementary material S4- Bioactivity assays**

*Myzus* *persicae* – Antifeedant assay

Control (left), Treatment (right)

Nematicidal assay

Antifungal assay- Spore germination inhibition

Antifungal assay- Mycelium growth inhibition
