# Supplementary material for: Endophytic fungi from two isolated Bethencourtia palmensis populations: metabolomic and bioactivity fingerprinting
Source: Pest Manag Sci. 2026 Jul 17;82(12):11151–61. doi: 10.1002/ps.71108 (PMC13756819; doi:10.1002/ps.71108)
Supplement: Supplementary file 2 [file PS-82-11151-s003.docx]

| ***B. palmensis* isolate** | **GenBank Accession no.** | **Species match in NCBI** | **Query cover (%)** | **Identity (%)** | **GenBank Accession no.** |
| --- | --- | --- | --- | --- | --- |
|  |  |  |  |  |  |
| *Alternaria* sp. strain BPF4.1 | PV938357 | *Alternaria tenuissima* | 100 | 100.00 | OR506015.1 |
| *Alternaria* sp. strain BPF5 | PV938358 | *Alternaria tenuissima* | 100 | 100.00 | MZ678512.1 |
| *Alternaria* sp. strain BPF6 | PV938359 | *Alternaria alternata* | 100 | 98.86 | OQ719879.1 |
| *Alternaria* sp. strain BPT2 | PV938381 | *Alternaria destruens* | 100 | 97.51 | PQ060533.1 |
| *Alternaria* sp. strain BPT3 | PV938382 | *Alternaria tenuissima* | 100 | 100.00 | OR506015.1 |
| *Alternaria* sp. strain BPT4 | PV938383 | *Alternaria tenuissima* | 100 | 100.00 | OR506015.1 |
| *Alternaria* sp. strain BPF7 | PV938360 | [*Alternaria alternata*](https://blast.ncbi.nlm.nih.gov/Blast.cgi#alnHdr_2624150296) | 97 | 100.00 | OR857254.1 |
| *Alternaria* sp. strain BPH1 | PV938368 | *Alternaria alternata* | 99 | 100.00 | KT827269.1 |
| *Aspergillus* sp. strain SPH2 | PV935591 | [*Aspergillus cretensis*](https://blast.ncbi.nlm.nih.gov/Blast.cgi#alnHdr_2679899107) | 100 | 100.00 | PP385367.1 |
| *Aspergillus* sp. strain SPH9 | PV935592 | [*Aspergillus tubingensis*](https://blast.ncbi.nlm.nih.gov/Blast.cgi#alnHdr_1820506061) | 100 | 100.00 | MT186175.1 |
| *Aspergillus* sp. strain BPT13 | PV938386 | *Aspergillus brasiliensis* | 100 | 94.74 | MK951939.1 |
| *Aspergillus* sp. strain BPG5 | PV938365 | *Aspergillus eucalypticola* | 100 | 100.00 | OQ135173.1 |
| *Aspergillus* sp. strain BPG6.2 | PV938367 | *Aspergillus niger* | 98 | 96.21 | PQ272547.1 |
| *Aspergillus* sp. strain BPH6 | PV938372 | [*Aspergillus niger*](https://blast.ncbi.nlm.nih.gov/Blast.cgi#alnHdr_2980635401) | 98 | 97.14 | PV663056.1 |
| *Aspergillus* sp. strain BPM1 | PV938373 | [*Aspergillus niger*](https://blast.ncbi.nlm.nih.gov/Blast.cgi#alnHdr_1862734685) | 100 | 96.64 | MT672509.1 |
| *Aspergillus* sp. strain BPM2 | PV938374 | *Aspergillus niger* | 100 | 97.67 | OR435288.1 |
| *Aspergillus* sp. strain BPM3 | PV938375 | *Aspergillus niger* | 100 | 96.52 | OR428166.1 |
| *Aspergillus* sp. strain BPM5 | PV938376 | *Aspergillus eucalypticola* | 100 | 100.00 | OQ135173.1 |
| *Aspergillus* sp. strain BPM7.1 | PV938377 | [*Aspergillus parasiticus*](https://blast.ncbi.nlm.nih.gov/Blast.cgi#alnHdr_2035121918) | 100 | 85.95 | MZ158627.1 |
| *Aspergillus* sp. strain BPM9.2 | PV938378 | [*Aspergillus luchuensis*](https://blast.ncbi.nlm.nih.gov/Blast.cgi#alnHdr_2208956309) | 99 | 97.30 | ON004124.1 |
| *Aureobasidium* sp. strain BPH3 | PV938369 | [*Aureobasidium pullulans*](https://blast.ncbi.nlm.nih.gov/Blast.cgi#alnHdr_1886986831) | 100 | 97.00 | MT862363.1 |
| *Aureobasidium* sp. strain BPH5 | PV938371 | [*Aureobasidium pullulans*](https://blast.ncbi.nlm.nih.gov/Blast.cgi#alnHdr_340400030) | 100 | 97.60 | HQ909088.1 |
| *Aureobasidium* sp. strain BPT1 | PV938380 | *Aureobasidium pullulans* | 100 | 99.55 | OK376900.1 |
| *Aureobasidium* sp. strain BPT15 | PV938388 | [*Aureobasidium pullulans*](https://blast.ncbi.nlm.nih.gov/Blast.cgi#alnHdr_2037009038) | 100 | 100.00 | MZ185334.1 |
| *Cladosporium* sp. strain BPM13 | PV938379 | [*Cladosporium cladosporioides*](https://blast.ncbi.nlm.nih.gov/Blast.cgi#alnHdr_1785470032) | 100 | 100.00 | MN844806.1 |
| *Epicoccum* sp. strain BPF3 | PV938356 | *Epicoccum nigrum* | 100 | 98.99 | KY617069.1 |
| *Mucor* sp. strain BPG3 | PV938364 | *Mucor racemosus* | 100 | 99.59 | LN809049.1 |
| *Penicillium* sp. strain SPH17 | PV935593 | [*Penicillium verrucosum*](https://blast.ncbi.nlm.nih.gov/Blast.cgi#alnHdr_1473259765) | 100 | 99.01 | MH872709.1 |
| *Penicillium* sp. strain BPH4 | PV938370 | *Penicillium glabrum* | 100 | 98.68 | MH854598.1 |
| *Penicillium* sp. strain BPG2 | PV938363 | [*Penicillium glabrum*](https://blast.ncbi.nlm.nih.gov/Blast.cgi#alnHdr_2185689742) | 96 | 97.40 | OM438160.1 |
| *Penicillium* sp. strain BPG6.1 | PV938366 | [*Penicillium glabrum*](https://blast.ncbi.nlm.nih.gov/Blast.cgi#alnHdr_2499336238) | 100 | 99.11 | OQ913811.1 |
| *Phoma* sp. strain BPT11 | PV938385 | *Phoma multirostrata* | 100 | 100.00 | KT013226.1 |
| *Stemphylium* sp. strain BPF8 | PV938361 | *Stemphylium vesicarium* | 100 | 98.56 | MT428427.1 |
| *Stemphylium* sp. strain BPF9 | PV938362 | [*Stemphylium gracilariae*](https://blast.ncbi.nlm.nih.gov/Blast.cgi#alnHdr_2032348262) | 98 | 98.32 | MZ093114.1 |
| *Stemphylium* sp. strain BPT9 | PV938384 | *Stemphylium eturmiunum* | 100 | 100.00 | MH843731.1 |

**Table S2.** GenBank accession numbers and closest BLAST matches in NCBI for ITS sequences of bioactive *B. palmensis* fungal isolates.
